# Recruitment and methodological issues in conducting dementia research in British ethnic minorities: A qualitative systematic review

**DOI:** 10.1002/mpr.1806

**Published:** 2019-12-05

**Authors:** Waquas Waheed, Nadine Mirza, Muhammed Wali Waheed, Amy Blakemore, Cassandra Kenning, Yumna Masood, Fiona Matthews, Peter Bower

**Affiliations:** ^1^ Division of Population Health, Health Services Research & Primary Care The University of Manchester Manchester UK; ^2^ Leicester Medical School University of Leicester Leicester UK; ^3^ Institute of Health and Society Newcastle University Newcastle UK

**Keywords:** barriers, BME, ethnic minorities, facilitator, solutions

## Abstract

**Objective:**

Identifying existing recruitment and methodological issues within dementia research conducted in UK studies that included ethnic minorities.

**Methods:**

We searched for and included any publication detailing dementia research in the UK that included any ethnic minority. The search results and all titles and abstracts were screened according to the inclusion criteria followed by screening of the full texts. We extracted data regarding the recruitment and methodological issues faced by the researchers. This data was combined and listed, and related issues were grouped into overarching themes and subthemes.

**Results:**

Of 52 publications suitable for analysis, 33 provided data collated into six themes: attitudes and beliefs about dementia in ethnic minority communities, recruitment process, data collection issues, practical issues, researcher characteristics, and lack of published research and normative data. These themes allowed us to identify three areas responsible for addressing these recruitment and methodological issues: community and patient education, health services, and researchers' training.

**Conclusions:**

This is the first review identifying recruitment and methodological issues within UK dementia research that included ethnic minorities. We now have a compilation of reported existing issues and a framework of areas responsible for addressing them and devising solutions.

## 1 INTRODUCTION

The ethnic minority population within the United Kingdom (UK) continues to increase, with present numbers accounting for 14% of the population (Office for National Statistics, 2016). Within these groups, there is an increasing ageing population, due to the timing of original migration and with more of the older population from these groups now choosing to permanently reside in the UK (Lievesley, 2010; Richards et al., 2000). Overall predictions show that by 2051, approximately 15–29% of ethnic minorities will be over the age of 65, a crucial age when considering dementia (Wohland, Rees, Norman, Boden, & Jasinska, 2010).

The overall diagnoses of dementia within the UK currently amounts to over 1.3% of the population, approximately 850,000 individuals (Prince et al., 2014). It is expected that this will increase to 1,000,000 by 2025, with one in three individuals now at risk of developing dementia within their lifetime (Dementia Statistics Hub , 2019).

Ethnic minorities account for 25,000 current dementia diagnoses (Department of Health, 2009), and this is expected to double by 2026 (Lakey, Chandaria, Quince, Kane, & Saunders, 2012). In fact, it is expected that the numbers are actually higher than reported, with predictions showing that while dementia overall will increase twofold within the next 40 years, it is expected to increase sevenfold within ethnic minorities (Alzheimer's Society, 2019).

This is due to a high susceptibility within ethnic minorities for receiving a diagnosis of dementia and higher rates of early onset dementia (Adelman, Blanchard, Rait, Leavey, & Livingston, 2011; Seabrooke & Milne, 2004). This is attributed to higher rates of the risk factors of developing dementia within ethnic minorities, such as diabetes, heart disease, and obesity (Adelman et al., 2011; Seabrooke & Milne, 2004).

Despite this, ethnic minorities are under‐represented in dementia research. The majority of studies do not target ethnic minorities, and researchers are not required in the UK to demonstrate ethnic minority representation within their research (Bhatnagar & Frank, 1997). Due to this, research designs do not account for issues specific to ethnic minorities within dementia research (Bhatnagar & Frank, 1997). This can range anywhere from their attitudes and beliefs about dementia (La Fontaine, Ahuja, Bradbury, Phillips, & Oyebode, 2007), which can prevent voluntary participation, to cultural and language barriers in the communication and assessment process and incompatible researcher characteristics, which negatively impacts all stages of research from recruitment to data analysis (Adelman et al., 2011; Shah, 1999; Turner, Christie, & Haworth, 2005). These issues are also coupled with a lack of expertise on addressing the issues as and when they arise (Adelman et al., 2011).

Therefore, including and retaining ethnic minorities to dementia research is further compromised, and therefore, any findings from such research cannot be applied to ethnic minorities or generalised to diverse populations. This inevitably leads to a paucity of literature on dementia research including ethnic minorities, with the majority of it having been conducted in the USA specific to their own ethnic minority populations (Adelman et al., 2011; Forbat, 2003). This, in turn, leaves future researcher's with little insight into UK‐based evidence on the dementia‐related numbers and needs of ethnic minorities and little guidance into what problems they may face in their own research and how to counter them. Thus, the cycle of lack of representation of ethnic minorities in dementia research continues.

This has implications for both UK‐based research and healthcare settings. As mentioned, results from existing dementia research are not always generalisable. In research being conducted, bias is not always eliminated, and ethical concerns, such as informed consent, arise (Richards & Abas, 1999). Alongside this, cognitive assessments developed through this research suffer from internal and external validity issues, resulting in higher rates of false positive and false negative scoring within ethnic minorities within clinical settings. We also find that the dementia diagnostic and care pathway, developed and implemented through this dementia research, cannot extend across cross‐cultural settings. The healthcare settings reflect their own bias and ethical concerns due to differences across ethnic minorities (Khan & Tadros, 2014; Regan, 2016; Weimer & Sager, 2009; Richards & Abas, 1999).

Therefore, ethnic minority representation within the context of UK‐based dementia research must be improved. This would require producing solutions to the problems researcher's currently face in recruiting, retaining, and including ethnic minorities within their dementia research. We propose that to do this there must first be a systematic understanding of the extent of the issues that currently exist and have been reported by researchers.

Previous reviews of the literature have explored issues UK researchers have faced including ethnic minorities within mental health research overall (Waheed, Hughes‐Morley, Woodham, Allen, & Bower, 2015). However, as yet, we have found no published review that details the issues that pertain specifically to dementia research. We propose identifying all existing recruitment and methodological issues within dementia research conducted in UK studies that included ethnic minorities. This would act as a precursor for devising potential solutions to negate each identified issue.

## 2 METHODS

We aimed to identify recruitment and methodological issues that arise when conducting dementia research that included British ethnic minorities. Therefore, eligible publications for this review included any publication detailing dementia research conducted in the UK, both qualitative and quantitative, that included any ethnic minority group. Studies that included UK dementia patients, family and carers, or healthcare professionals working with dementia patients were eligible for inclusion, and we did not exclude publications based on methodology or year of publication. Published dissertations were included, but unpublished dissertations and conference proceedings were not.

The guidelines on the reporting of systematic reviews in the PRISMA statement were followed (Moher, Liberati, Tetzlaff, & Altman, 2009).

### 2.1 Search strategy

The search strategy was devised within the team (CK, AB, and WW; Blakemore et al., 2018) to identify all studies related to dementia and ethnicity where ethnicity referred to the specific UK‐based ethnic minority groups as reported across the 2011 Census (Office for National Statistics, 2016). This included any ethnic group that was not classified as White British, where Irish and Welsh were also considered as minorities for the purposes of this review.

Broad search terms were purposefully chosen to maximise the number of studies that were eligible for inclusion. Search terms included “dementia,” “Alzheimer*,” “ethnic*,” “Asian,” “Black,” “African,” “minority,” “ethnic group,” and “multiethnic.” The search was conducted without a study design filter to allow for the retrieval of both qualitative and quantitative studies.

We searched the Cochrane Register of Controlled Trials, EMBASE, PsychINFO, MEDLINE, and the Cochrane Database of Systematic Reviews. The search was initially run till June 2017, with the earliest year being 1860, and then updated till June 2018. We also hand searched the reference lists of all relevant systematic and literature reviews.

### 2.2 Data screening and extraction

The search results were exported to EndNote X7 (Clarivate Analytics, Philadelphia, PA). All titles and abstracts were screened by AB according to the inclusion criteria set out. This was followed by screening of the full texts by NM, which were done by hand. When full texts of potentially eligible publications were not available, authors were contacted to request the paper. Ambiguities regarding the inclusion of a publication were discussed by WW and NM.

We extracted data from the full texts of all eligible papers regarding the recruitment and methodological issues faced by the researchers. We used a data extraction sheet that had been developed a priori and pilot tested. Data was extracted by two independent reviewers, NM and MWW.

The data extraction sheet firstly extracted descriptive information about the first author, the site of the research, and the ethnicities included. In addition to this, it also extracted data on the following recruitment and methodological processes: the recruitment process of interviewers, cultural considerations within interviewer training, ethnic matching of interviewers, method of recruitment and obtaining consent, the provision of incentives for participants, the method of assessing literacy and age, the method of administering any additional assessments, considerations when selecting assessments, languages offered, process of translation, project promotion within relevant communities, process of engaging with families, and any other barriers identified.

### 2.3 Data analysis and synthesis

The data was analysed and synthesised according to procedures relayed by Braun and Clarke (2008) for thematic analysis. The data on recruitment and methodological issues extracted from all included publications were combined to create a full list of mutually exclusive extracts hereafter referred to as codes.

Using the informal approach of cutting and sorting (Ryan & Bernard, 2003), seemingly related codes were clustered by WW, NM, and MWW (Speer & McPhillips, 2013). Themes were then formulated according to what the clusters were describing, with no minimum limit on the number of codes per theme. As the importance of themes was considered, some were merged into one, and some were grouped and became subthemes, linked under an overarching theme. Some codes were shared by more than one theme or subtheme, highlighting the relations between individual themes overall.

The themes and subthemes were then revised and refined and given labels to signify what they represented (Braun & Clarke, 2008).

## 3 RESULTS

The search identified 6,481 papers, of which 6,361 were excluded by removing duplicates and screening the titles and abstracts against the eligibility criteria. The full texts of the remaining 120 papers were screened by hand, of which 68 were excluded for being systematic or other literature reviews, not reporting primary data, not from the UK, or not focused on dementia.

**Figure 1:**
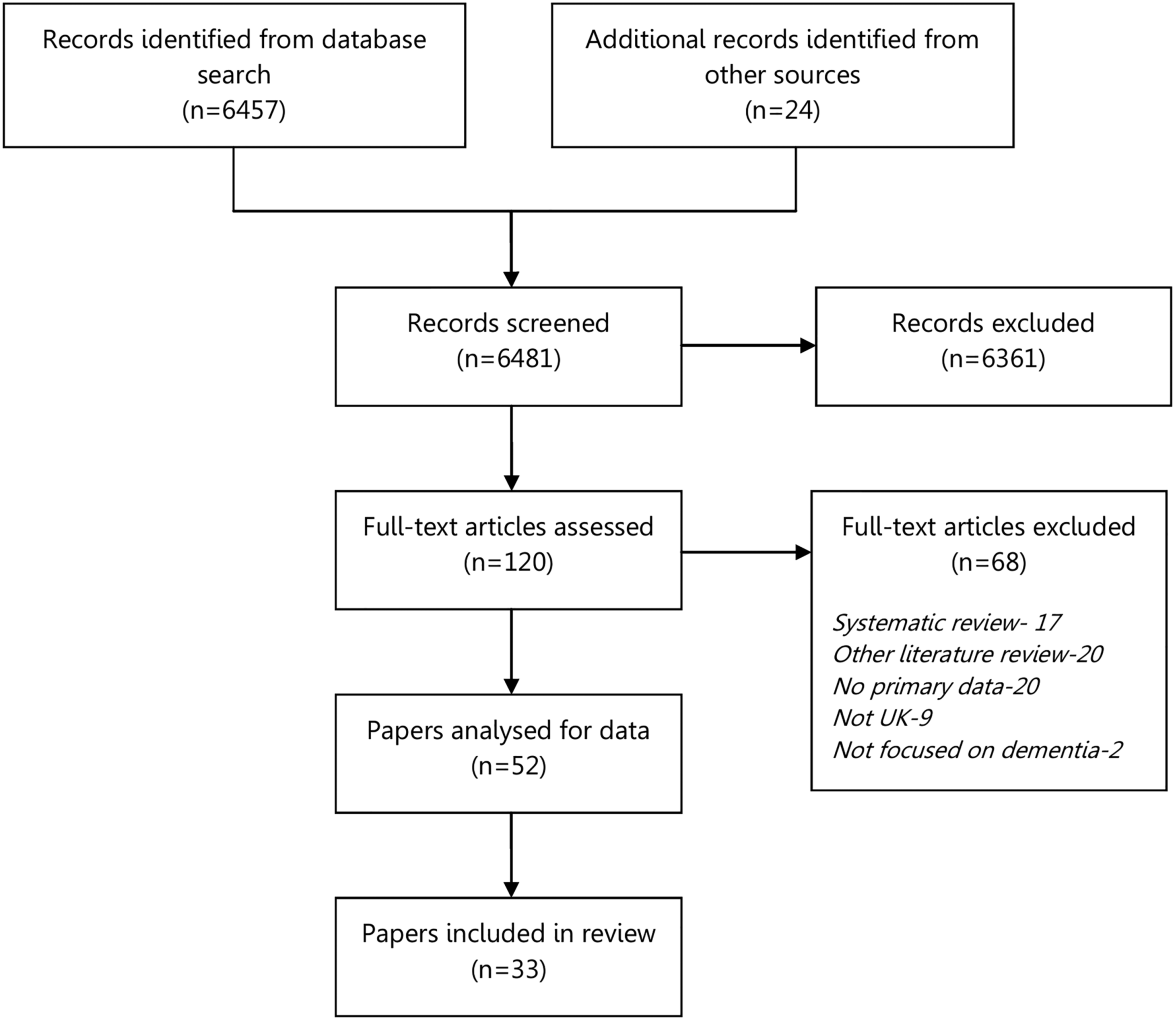
Flow diagram of paper selection

The remaining 52 papers were all in English, detailing dementia research that included ethnic minorities in the UK. Across these papers, 9 were from 1995 to 2000, 14 from 2000 to 2005, 13 from 2005 to 2010, 8 from 2010 to 2015, and 8 from 2015 onwards. Of these 52 papers, 33 described methodological issues faced by the researchers. A flow diagram of the paper selection is provided in Figure 1. Of these papers, 23 included South Asians, 12 included Afro‐Caribbeans, 3 included Chinese, and 2 included Eastern Europeans. Ethnic minorities identifying as Creole, Greek Guyanese, Japanese, Polish, Turkish, and Welsh were each included in one paper. A summary of these papers is provided in Table 1.

**Table 1:** Characteristics of the analysed papers

Study	Setting	Ethnic group	Design	Sample size	Description
Adamson, 2001 [37]	East Midlands, North West, South East, and South West	Afro‐Caribbean and South Asian	Qualitative study (semistructured interviews)	30	Exploring awareness and understanding of dementia symptoms in families of Afro‐Caribbean and South Asian descent through semi‐structured interviews
Adamson & Donovan, 2005 [44]	London, Bristol, Leicester, Bradford, Nottingham	Afro‐Caribbean and South Asian	Exploratory qualitative study (semistructured interviews)	21	Examining the experience of caring for an older family member, focusing on Afro‐Caribbean and South Asian carers of a person with dementia through semi‐structured interviews
Adelman et al., 2011 [8]	5 general practices in North London	Afro‐Caribbean	Cross‐sectional prevalence study	436	Determining the prevalence of dementia in older people of Afro‐Caribbean country of birth compared with their White UK‐born counterparts
Beattie, Daker‐White, Gilliard, & Means, 2005 [22]	Local authority areas in South West England	Afro‐Caribbean	Field study (interviews)	61	Examining the accessibility of dementia services for people under 65 and people from minority ethnic groups through interviews and assessing the main issues in service access
Bhatkal & Shah, 2004 [24]	Geriatric psychiatry services in West London	Polish	Qualitative study (case studies)	321	Comparing the clinical, demographic, social, and service utilisation characteristics of elderly patients of Polish origin newly referred to a geriatric psychiatry service
Bhatnagar & Frank, 1997 [12]	Bradford	South Asian	Community study	213	Assessing the prevalence of psychiatric disorders amongst elderly South Asian immigrants from the Indian subcontinent living in Bradford, UK
Bowes & Wilkinson, 2003 [33]	Urban Scottish settings	South Asian	Qualitative study (case studies and interviews)	11	Examining the view and experiences of dementia amongst older South Asian people, their families and carers, and to explore central issues of service support through interviews and case studies
Haider & Shah, 2004 [25]	Geriatric psychiatry service in West London	South Asian	Pilot study	59	A study of behavioural and psychological signs and symptoms of dementia in patients of Indian subcontinent origin admitted to a dementia day hospital in the UK
Hailstone, Mukadam, Owen, Cooper, & Livingston, 2017 [40]	London	South Asian	Qualitative study (focus groups and interviews)	58	Devising and validating a theory of planned behaviour questionnaire to measure attitudes to help‐seeking for dementia in people from South Asian backgrounds in the UK to assess the effectiveness of future interventions
Jutlla, 2015 [26]	Wolverhampton	South Asian	Qualitative study (narrative interviews)	12	Exploring how migration experiences and life histories impact on perceptions and experiences of caring for a family member with dementia using narrative interviews
La Fontaine et al., 2007 [13]	Birmingham	South Asian	Qualitative study (focus groups)	49	Exploring perceptions of ageing, dementia, and ageing‐associated mental health difficulties amongst British people of Punjabi origin through focus groups
Lawrence, Murray, Samsi, & Banerjee, 2008 [45]	South London boroughs	Afro‐Caribbean and South Asian	Qualitative study (interviews)	32	Exploring the caregiving attitudes, experiences, and needs of family carers of people with dementia from Afro‐Caribbean, South Asian, and White backgrounds through interviews
Livingston et al., 2001 [36]	Islington, North London	Afro‐Caribbean, Turkish, and Greek	Cross‐sectional community study	1085	Comparing the prevalence of dementia and depression in older migrants with those born in the UK through a cross‐sectional community study
Mackenzie, 2006 [23]	Bradford	South Asian and East European	Qualitative study (semistructured interviews)	11	Exploring carers' experiences of caregiving, the nature and availability of family, community, and mainstream service support, knowledge of dementia, and what the carers would want from a support‐group programme through semistructured interviews
McCracken et al., 1997 [27]	Liverpool inner city	Afro‐Caribbean, Chinese, and South Asian	Qualitative prevalence study (interviews)	418	Identifying all elderly people of ethnic minorities living in a defined geographical area in inner‐city Liverpool and to identify psychiatric morbidity and barriers to use of services through interviews
Morgan & Crowder, 2003 [28]	Wales	Welsh	Quantitative repeated measures study	31	Determining whether Welsh‐speaking clients suffering from dementia scored differently when assessed using the MMSE in English and in Welsh
Mukadam, Cooper, Basit, & Livingston, 2011 [29]	London inner city	Afro‐Caribbean, Chinese, and South Asian	Qualitative study (semistructured interviews)	18	Determining the barriers to and facilitators of help‐seeking and the pathways to diagnosis through semistructured interviews
Odutoye & Shah, 1999 [30]	West Middlesex University Hospital	South Asian	Qualitative study (case studies)	242	Comparing the clinical, demographic, social, and service‐utilisation characteristic between ethnic elders of Indian subcontinent origin and indigenous elders through case studies
Parveen, Blakey, & Oyebode, 2018	9 sites across UK	South Asian	Qualitative study (focus groups and interviews	59	Evaluating a carer's information programme culturally adapted for South Asian families
Purandare, Luthra, Swarbrick, & Burns, 2007 [34]	Manchester	South Asian	Quantitative study (questionnaires)	255	Examining the knowledge of dementia in South Asian older people as compared to with Caucasian older people, through the Dementia Knowledge Questionnaire
Rait et al., 2000 [38]	Manchester inner city	Afro‐Caribbean	Validation study	130	Developing culturally modified versions of the MMSE and Abbreviated Mental Test and determining their sensitivity and specificity by comparing them with a diagnostic computerised interview
Rait et al., 2000 [39]	Central Manchester	South Asian	Validation study	120	Developing culturally modified versions of the MMSE and Abbreviated Mental Test and determining their sensitivity and specificity by comparing them with a diagnostic computerised interview
Ramsey, Stevens, Bryan, Binder, & Cockle‐Hearne, 2009 [41]	Surrey	Chinese, Creole, European, Guyanese, and Japanese	Validation study	144	Tested the hypothesis that those with English as an additional language would score less well than those with English as a first language on the sub‐tests of the Barnes Language Assessment and elucidate the reasons for any such differences and discuss the implications.
Redelinghuys & Shah, 1997 [35]	Geriatric psychiatry service, West London	South Asian	Cross‐sectional comparative study	235	Examining the demographic, social, and clinical characteristics of Indian subcontinent origin elders with mental illness through a cross‐sectional comparative study
Regan, 2016 [18]	London	South Asian	Qualitative study (case studies, interviews, and observations)	1	Investigating the motivations and experiences of accessing dementia care health and social care services for a Muslim, Pakistani male with dementia through a case study comprising formal interviews and observations and informal discussions
Richards et al., 2000 [3]	Southwark, London borough	Afro‐Caribbean	Pilot study	86	Investigating cognitive function in UK community dwelling Afro‐Caribbean and White elders
Shah, 1999 [14]	London	South Asian	Qualitative study (interviews)	12	Exploring difficulties experienced by a Gujarati psychiatrist in interviewing Gujarati elders in Gujarati
Shah, Lindesay, & Jagger, 1998 [43]	Leicester	South Asian	Qualitative study (interviews and surveys)	11	Establishing the stability of an original dementia diagnosis over time in elderly Gujaratis through original cross cultural surveys and reinterviews
Stewart, Richards, Brayne, & Mann, 2001 [42]	South London community primary care practices	Afro‐Caribbean	Cross‐sectional normative data study	285	To describe normative data for cognitive function in UK community‐dwelling Afro‐Caribbean elders
Stewart et al., 2001 [46]	South London primary care	Afro‐Caribbean	Community‐based study	302	Examining the association between APOE genotype and early cognitive impairment in a community‐based Afro‐Caribbean UK population
Turner et al., 2005 [15]	South London boroughs, Merton, and Wandsworth	South Asian	Qualitative study (semistructured interviews)	192	Discovering whether there were differences in views about the nature, causes, and treatments for dementia and who participants believed should provide care between South Asian and White older people
Uppal, Bonas, & Philpott, 2014 [31]	Sikh gurdwara in East Midlands	South Asian	Qualitative study (focus groups)	28	Exploring the understanding and perceptions of dementia amongst Sikhs living in the UK through focus groups
Wilkinson, Bowes, & Rodrigues, 2003 [32]	Urban Scottish settings	South Asian	Feasibility study (case studies and interviews)	11	Examining the methods used in a feasibility study that aimed to find ways of making contact with and gaining access to people from South Asian communities with a diagnosis of dementia and to explore their experience of service provision

Abbreviations: APOE Apolipoprotein E; MMSE Mini‐Mental State Examination.

The recruitment and methodological issues identified were collated into six themes, with their own individual subthemes: attitudes and beliefs about dementia in the ethnic minority communities, recruitment process, data collection issues, practical issues, researcher characteristics, and lack of published research and normative data. It is important to note that the themes were found to be interrelated and can never be truly mutually exclusive due to similar and overlapping concepts. The frequency of these themes across the papers is provided in Table 2.

**Table 2:** Frequency of recruitment and methodological issues across the papers

Themes	Subthemes	Adamson, 2001	Adamson & Donovan, 2005	Adelman et al., 2011	Beattie et al., 2005	Bhatkal & Shah, 2004	Bhatnagar & Frank, 1997	Bowes & Wilkinson, 2003	Haider & Shah, 2004	Hailstone et al., 2017	Jutlla, 2015	La Fontaine et al., 2007	Lawrence et al., 2008	Livingston et al., 2001	Mackenzie, 2006	McCracken et al., 1997	Morgan & Crowder, 2003	Mukadam et al., 2011	Odutoye & Shah, 1999	Parveen et al., 2018	Purandare et al., 2007	Rait, Burns, et al., 2000	Rait, Morley, et al., 2000	Ramsey et al., 2009	Redelinghuys & Shah, 1997	Regan, 2016	Richards et al., 2000	Shah, 1999	Shah et al., 1998	Stewart, Richards, et al., 2001	Stewart, Russ, et al., 2001	Turner et al., 2005	Uppal et al., 2014	Wilkinson et al., 2003
1) Attitudes and beliefs about dementia in ethnic minority communities					X		X					X			X																			
2) Recruitment process																																		
	a) Diagnostic label of dementia				X	X		X	X		X	X				X	X	X	X		X					X		X					X	X
	b) Issues defining and identifying ethnicities	X				X			X			X		X		X			X			X	X		X			X						
3) Data collection issues																																		
	a) Language issues in assessments						X			X				X						X				X				X						
	b) Cultural issues in assessments			X			X																				X	X		X				X
	c) Translation of assessments											X											X					X						
	d) General communication issues	X	X				X					X		X	X			X						X				X	X					X
4) Practical issues				X	X		X	X												X														
5) Researcher characteristics							X					X																X				X		
6) Lack of published research and normative data													X		X											X		X		X	X	X		

**Figure 2:**
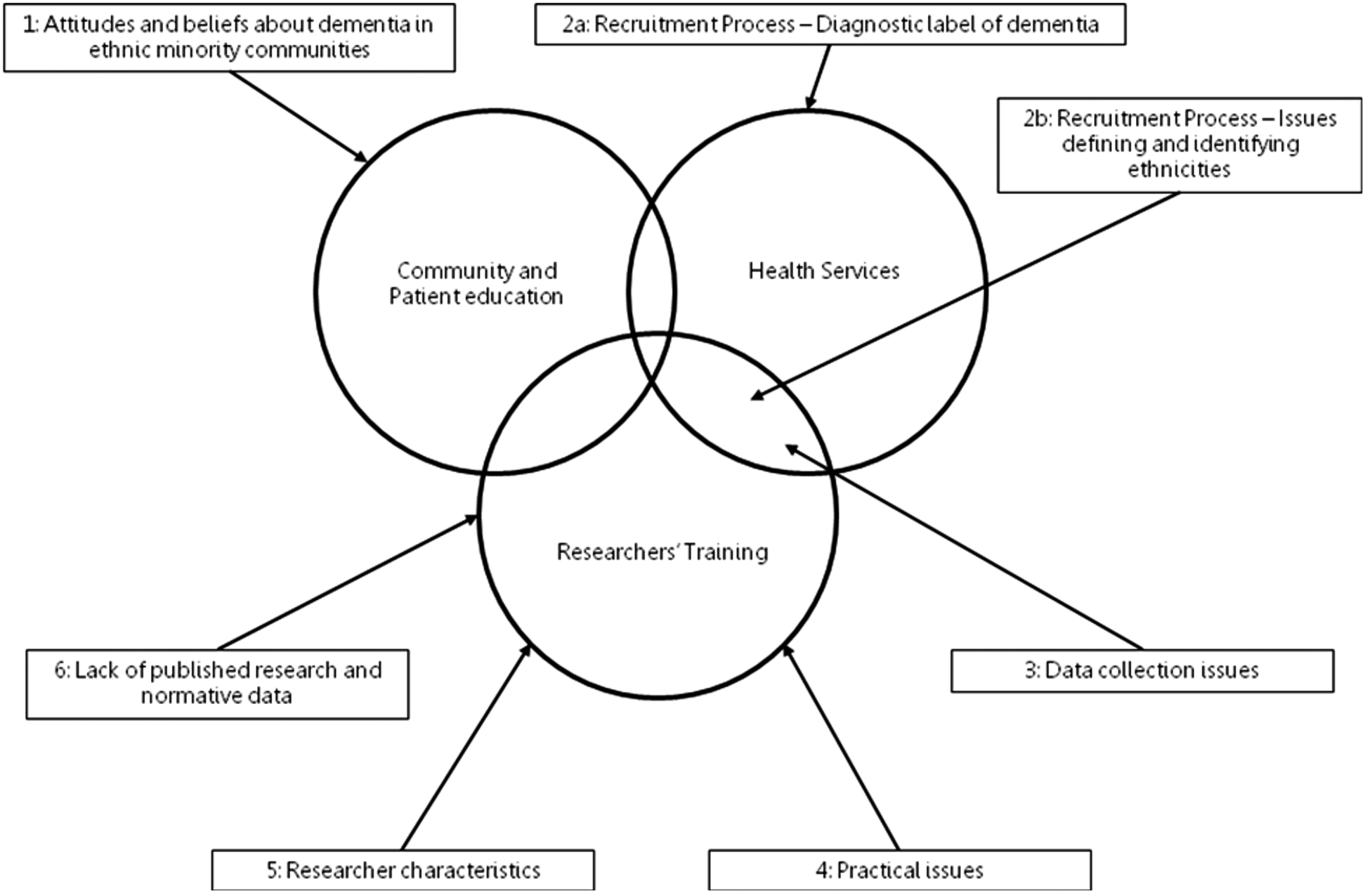
Areas responsible for addressing issues identified

These structured themes allowed us to identify three areas requiring intervention to address and improve the recruitment and methodological issues the themes represent, community and patient education, health services, and researchers' training. Due to the themes and subthemes being interrelated, we found that one area may be responsible for more than one research or methodological issue and that a single issue may be addressed by more than one area. These three areas have been represented in a Venn diagram in Figure 2, with directional arrows indicating which theme, as well as respective subthemes, should be addressed by which area or areas.

### 3.1 Attitudes and beliefs about dementia in ethnic minority communities

Certain attitudes and beliefs about dementia and receiving a diagnosis resulted in individuals from various ethnic communities being reluctant to contribute to or participate in dementia research. This reluctance was attributed to stigma within these communities regarding dementia and mistaking it for the natural progression of ageing.

Three papers reported the impact of stigma and discrimination related to dementia within Afro‐Caribbean, East European, and South Asian communities that deters participation. This was ascribed to the shame individuals are made to feel when their diagnosis of dementia is revealed within their families and communities (Bhatnagar & Frank, 1997; La Fontaine et al., 2007; Mackenzie, 2006).

One paper reported that within Afro‐Caribbean communities, a diagnosis of dementia is perceived as unimportant because there are prevailing beliefs that symptoms of dementia are a consequence of regular ageing (Beattie et al., 2005). This diminishes the importance of addressing symptoms of dementia when also dealing with other more overt concerns, both social and health related (Beattie et al., 2005).

### 3.2 Recruitment process

Authors that conducted dementia research within South Asian communities described the recruitment process to be “notoriously challenging” (Regan, 2016) and requiring “many visits and a high degree of persistence” (Bhatnagar & Frank, 1997). This was found to be the case across other ethnic minorities as well and has been attributed to the subthemes of “determining a diagnostic label of dementia” and “issues defining and identifying ethnicities.”

#### 3.2.1 Diagnostic label of dementia

Authors stated that they faced difficulty recruiting participants with a diagnosis of dementia from ethnic minorities because these populations are already underrepresented within health services (Regan, 2016).

Eleven papers brought up the issue of there being a limited catchment area in terms of population numbers to recruit Afro‐Caribbean, Chinese, and South Asian participants from (Beattie et al., 2005; Bhatkal & Shah, 2004; Haider & Shah, 2004; Jutlla, 2015; La Fontaine et al., 2007; McCracken et al., 1997; Morgan & Crowder, 2003; Mukadam et al., 2011; Odutoye & Shah, 1999; Uppal et al., 2014; Wilkinson et al., 2003). Three papers noted that when an Afro‐Caribbean or South Asian sample was identified, potential participants often did not have a formal diagnosis. Authors stated that this was because clinicians, such as psychiatrists, are trained solely in the English language (Shah, 1999), creating an inevitable language barrier, and lack the cultural knowledge that would allow them to communicate with patients clearly enough to form a necessary diagnosis (Beattie et al., 2005; Bowes and Wilkinson et al., 2003). One paper also noted that South Asians perceive a dementia diagnosis to be a sensitive matter they may not wish to disclose to researchers, linking to the theme of “attitudes and beliefs about dementia in ethnic minority communities,” making identifying those with dementia a timely and difficult process (Wilkinson et al., 2003).

One paper also cited that potential participants may have been misdiagnosed with dementia as there is a “historical and pervasive racism” that can lead to ethnic minorities being mislabelled with dementia despite having a different health problem (Beattie et al., 2005). Another also highlighted that researchers may draw conclusions when they are not blind to the ethnicity of participants (Haider & Shah, 2004).

On the other hand, one paper reported that recruiting healthy participants from a South Asian sample becomes compromised due to the high rates of those undiagnosed with dementia (Purandare et al., 2007). This can once again be attributed to not identifying symptoms of dementia and therefore not receiving a diagnosis, as mentioned in the theme of attitudes and beliefs about dementia in ethnic minority communities. This is also affected by problems that arise within the diagnostic cognitive assessment process, discussed in the theme “Data Collection.”

#### 3.2.2 Issues defining and identifying ethnicities

Three papers stated that both “the definition and identification of ethnic minority patients can pose difficulties” (Bhatkal & Shah, 2004; Odutoye & Shah, 1999; Redelinghuys & Shah, 1997).

Six papers focused on the difficulties that arise when attributing an individual to a particular ethnicity based on a standard classification (Haider & Shah, 2004) or an individual's broad background, be it Afro‐Caribbean, Chinese, Greek, South Asian, or Turkish (Haider & Shah, 2004; Livingston et al., 2001; McCracken et al., 1997; Odutoye & Shah, 1999; Redelinghuys & Shah, 1997; Shah, 1999).

It results in the misclassification of individuals due to the heterogeneity that exists even within ethnic minorities, with subgroups differing within themselves in terms of many facets including language, culture, and religion (Haider & Shah, 2004; Livingston et al., 2001). This was seen particularly in British South Asian individuals, who are often grouped together, despite the diversity across individual subgroups, thus making the research results “unrepresentative or meaningless” (La Fontaine et al., 2007; Odutoye & Shah, 1999; Shah, 1999). .

Four papers then described how identifying Afro‐Caribbean, Chinese, or South Asian patients from lists and registers for research purposes was “challenging and time consuming” (Adamson, 2001; McCracken et al., 1997;Rait, Burns, et al., 2000 ; Rait, Morley, et al., 2000). Reasons for this include there being no practice registers specifically for ethnic minority patients (Rait, Burns, et al., 2000; Rait, Morley, et al., 2000), that not all ethnic minority individuals are registered with a GP (McCracken et al., 1997), and that even when they are on these lists it can be difficult to recognise ethnic minority forenames and surnames, particularly those that are Afro‐Caribbean (McCracken et al., 1997; Rait, Morley et al.).

### 3.3 Data collection issues

#### 3.3.1 Language issues in assessments

Six papers discussed how Afro‐Caribbean, Greek, South Asian, and Turkish participants may struggle to complete assessments that have a heavy reliance on English and are dependent on literacy, such as cognitive assessments (Bhatnagar & Frank, 1997; Hailstone et al., 2017; La Fontaine et al., 2007; Livingston et al., 2001; Parveen et al., 2018; Ramsey et al., 2009). Older people from ethnic minority backgrounds, who are already limited by language and rely on others to translate, are also only likely to mix with those from the same ethnic and language background. This would give them a limited need and opportunity to learn or improve upon their English (Ramsey et al., 2009).

One paper reflected how only their English‐speaking South Asian participants could complete the Dementia Knowledge Questionnaire (Hailstone et al., 2017), and another ruled out self‐administered questionnaires entirely because of linguistic issues (Parveen et al., 2018). A third paper stated that it was difficult to assess the cognitions of their participants as they all spoke Gujarati and only two could write English (Shah, 1999). One paper stated how the only solution would be to have validated versions of such assessments in South Asian languages, such as Punjabi, Gujarati, and Bengali, but they did not exist at the time of their research (Bhatnagar & Frank, 1997).

#### 3.3.2 Cultural issues in assessments

Six papers reflected how, not just language, but culture also causes issues in assessments with Afro‐Caribbean and South Asian participants as they often express a preference for, or reinforcement of, the culture of their home country (Adelman et al., 2011; Bhatnagar & Frank, 1997; Richards et al., 2000; Shah, 1999; Stewart, Richards, et al., 2001; Wilkinson et al., 2003). One paper attributed this issue in an Afro‐Caribbean older population to their education being “Caribbean culture based”, therefore leaving them unfamiliar with Western culture (Richards et al., 2000). Despite this, there is a lack of fully validated culturally adapted assessments across ethnicities, as highlighted by one paper (Wilkinson et al., 2003).

Therefore, participants struggle with questions and tasks commonly seen in cognitive assessments utilised to diagnose dementia, such as providing birth or relevant dates due to following calendars other than the Western calendar (Shah, 1999) and demonstrating issues with name spelling and both historical and general knowledge. One paper stated that South Asian participants could not name the UK Prime Minister but could name South Asian leaders from the countries they had originally migrated from (Bhatnagar & Frank, 1997).

Such cultural bias has resulted in false positives and negative results when using screening and diagnostic assessments with South Asian and Afro‐Caribbean populations in any context (Adelman et al., 2011; Bhatnagar & Frank, 1997).

#### 3.3.3 Translation of assessments

Authors that worked with Eastern European and South Asian participants attempted to address the above issues by translating assessments themselves, but they faced a myriad of issues. One paper detailed that there were no standard guidelines on translating any given assessment (Rait, Burns, et al., 2000) and even when a translation was managed there were no back translations to act as a check (La Fontaine et al., 2007).

One paper cited that many assessment questions pertaining to psychiatric and psychological symptoms were difficult to translate from English into Gujarati due to an absence of the necessary vocabulary in the Gujarati language (Shah, 1999). This also presents in the below subtheme “General communication issues.”

#### 3.3.4 General communication issues

Authors cited that there were communication issues throughout different stages of their dementia research.

Four papers attributed language barriers as an issue for general communication beyond the use of assessments mentioned above (Livingston et al., 2001; Rait, Burns, et al., 2000; Shah, 1999; Shah et al., 1998). Older people from Afro‐Caribbean, Chinese, and South Asian communities were more likely to not have English as a first language or be monolingual (Livingston et al., 2001; Ramsey et al., 2009; Shah, 1999). On the other hand, two papers acknowledged that the authors themselves did not speak any South Asian languages to aid them in general communication, which also links to the below theme “researcher Characteristics” (Adamson, 2001; Adamson & Donovan, 2005).

Two papers also cited instances of illiteracy causing communication issues with South Asian participants who could not read or write in their mother tongue and had never made use of a pen or pencil either (Bhatnagar & Frank, 1997; Shah, 1999).

Two papers reported that the materials used throughout their dementia research were only available in English, such as transcripts that required comments from Afro‐Caribbean, Chinese, and South Asian participants (Mukadam et al., 2011; Shah et al., 1998). Furthermore, clinical interview schedules that are used to take informant history from potential participants, such as from Gujarati‐speaking South Asians, were not available in target languages like Gujarati (Shah et al., 1998). This gap in language between the researcher and ethnic minority population can result in participants being unable to report other health conditions, especially those relevant to mental health (Shah, 1999).

One paper highlighted the importance of cultural understanding within conversation and how health professionals' lack of knowledge of South Asian culture hindered communication (Wilkinson et al., 2003).

The issues highlighted with general communication resulted in many authors requiring the use of interpreters throughout their research (La Fontaine et al., 2007). Two papers reflected on how interviews with Afro‐Caribbean and South Asian populations that incorporated interpreters; they resulted in stilted and unnatural conversations that made the data collected less rich due to nuances that were lost, which could otherwise have been identified by researchers who were familiar with and could speak the language (Adamson, 2001; Adamson & Donovan, 2005). This once again speaks to the below theme researcher Characteristics as well.

Interpreters were also unable to address the fact that South Asians do not have a word for dementia in their languages, with any closely related words being considered vague and disrespectful (Mackenzie, 2006; Wilkinson et al., 2003). This links back to the concept of limited vocabulary, mentioned in the subtheme “Translation of assessments.”

Two papers also described the presence of other family members, who would not leave during sessions, as making communication and information gathering more difficult with South Asian participants as opposed to bridging the gap (Shah, 1999; Wilkinson et al., 2003).

#### 3.3.5 Practical issues

Four papers cited a variety of practical issues they encountered across Afro‐Caribbean and South Asian communities throughout the recruitment and methodological processes (Adelman et al., 2011; Beattie et al., 2005; Bhatnagar & Frank, 1997; Bowes & Wilkinson, 2003).

Lack of financial resources was a recurrent theme across both participants and researchers. Authors of research with Afro‐Caribbean participants noted there is not enough funding for research within marginalised groups, including ethnic minorities (Beattie et al., 2005). Research with South Asian participants demonstrated a burden on finances and time, due to the increased involvement of identifying, contacting, and building a rapport with their potential participants, all efforts that occur before the research even begins (Bowes & Wilkinson, 2003).

On the other hand, ethnic minorities are more likely to have a low socioeconomic status. Therefore, they may not have the time or resources to allow for their participation, especially voluntary, such as child minders or means of transport, to allow them to participate in dementia research (Adelman et al., 2011).

Three papers also discussed the issue of participant availability amongst Afro‐Caribbeans and South Asians, even after recruitment (Adelman et al., 2011; Bhatnagar & Frank, 1997; Parveen et al., 2018). One author noted that participants were not always able to commit sufficient time to research due to other responsibilities and were therefore reluctant when completing cognitive assessments as they were considered “lengthy” (Adelman et al., 2011). Two others spoke of the problem of participant retention and loss of follow‐up (Parveen, Blakey and Oyebode), stating that one of the reasons was that participants were very likely to demonstrate “high mobility”, which included visiting their home countries to fulfil obligations of meet with relatives (Bhatnagar & Frank, 1997).

### 3.4 Researcher characteristics

Three papers discussed the discrepancy in cultural background between researchers and participants as an issue in conducting dementia research within South Asian communities (Bhatnagar & Frank, 1997; Shah, 1999; Turner et al., 2005). This exploration links to but also goes beyond the previous subtheme of General communication issues.

Papers found that within qualitative research especially, a discrepancy in culture and background impacts and restricts the analysis of data. Raters and interviewers with a non‐South Asian background, most likely Western, struggled to interpret questionnaire data in one paper (Shah, 1999) and the analysis of interview transcripts in another (Bhatnagar & Frank, 1997). This occurs due to “differences … in the assumptions and world views of the researchers and participants” (Turner et al., 2005).

However, one paper also brought up the issue of recruiting researchers from within ethnic communities, relating to the theme of attitudes and beliefs about dementia in ethnic minority communities (La Fontaine et al., 2007). Potential British South Asians participants were reluctant to work with a researcher of their own ethnicity due to a fear that their information or data may be exposed within their close‐knit communities (La Fontaine et al., 2007). This often demonstrates confusion around the concept of confidentiality within research.

One paper also reported how a significant age difference between the researcher and participant can cause difficulties in conducting research due to the cultural significance in South Asia of elders being in a position of power and knowledge and younger individuals deferring to them (Shah, 1999).

### 3.5 Lack of published research and normative data

Seven papers highlighted the current lack of dementia‐related literature regarding ethnic minorities, in particular Afro‐Caribbean, Eastern European, and South Asian communities within the UK. (Lawrence et al., 2008; Mackenzie, 2006; Regan, 2016; Shah, 1999; Stewart, Richards, et al., 2001; Stewart, Russ, et al., 2001; Turner et al., 2005).

This is primarily regarding normative data of cognitive tests to compare against (Shah, 1999; Stewart, Richards, et al., 2001; Stewart, Russ, et al., 2001) though one paper detailed that this also extends beyond statistics to issues of diagnosis, identifying and contacting respondents, social identity, and gaining informed consent, all of which would be beneficial literature to future dementia researchers (Regan, 2016).

## 4 DISCUSSION

This is the first systematic review of its kind to identify the recruitment and methodological issues within dementia research that has been conducted in the UK with ethnic minorities. The benefit of this is that we now have a compilation of all of the existing issues across different research stages that have been reported by both participants and researchers.

As this review accounts for issues reported within published literature, it is limited by not being able to account for issues that may have arisen but were not reported by the authors. This explains why we were able to identify 52 publications that discussed UK‐based dementia research with ethnic minorities, but why 19 of these did not report issues. The data reported across publications was also condensed to suit write‐ups and may have lacked additional information. A review that explored global literature would have potentially yielded more papers and therefore more data. However, it was crucial to focus specifically on the UK's specific communities and healthcare system and research within it and to identify issues that pertain to the particular ethnic minorities residing in the UK.

Despite this, our findings highlight the barriers that emerge not just during the research process but prior to it across a range of ethnicities. South Asians demonstrated the most common ethnicity explored within the literature, which may be due to them being the UKs largest ethnic minority group (Office for National Statistics, 2016). However, it may also be indicative of a lack of representation of other ethnic minority groups in the already limited dementia research that includes ethnic minorities. We can see that in 5‐year increments, there has been a downward trend in UK‐based dementia research including ethnic minorities. All papers included also described research with the specific intent to recruit from a particular ethnic minority group. This highlights a lack of overall dementia research being conducted within diverse UK populations that happen to also include ethnic minorities, or that the inclusion of ethnic minorities is simply not being reported.

The themes uncovered extended from the initial stage of recruitment to the analysis of collected data and demonstrated a dual nature: issues in dementia research within any target population, or issues concerning any type of research conducted with ethnic minorities. For example, certain issues, such as a lack of funding for research within marginalised groups, are not restricted to ethnic minorities and are applicable to any groups that identify as being from a low socioeconomic background (Brown, Marshall, Bower, Woodham, & Waheed, 2014). In the same way, issues such as a stigma of mental health illness within ethnic minority populations exist beyond dementia to research in other mental health problems (Brown et al., 2014). Issues such as a lack of appropriately translated and culturally adapted health measures and language barriers between a researcher and participants also extends beyond mental health research, to research in physical health problems (Beaton, Bombardier, Guillemin, & Ferraz, 2000).

This review, as mentioned, also allowed us to determine the three areas responsible for addressing these issues, with different issues pertaining to the different areas, community and patient education, health services, and researcher training. We see that the barrier of attitudes and beliefs about dementia in ethnic minority communities requires changes and amendments made to the current quality of dementia‐related education, with ethnic minority patients and within their communities. On the other hand, the issue of the “diagnostic label of dementia” must be addressed by health services.

Across recruitment and methodological issues, it can be seen that they were very much interrelated, with connections made throughout themes. Therefore, we also see certain issues overlapping within areas responsible for them, such as issues defining and identifying ethnicities, which both health services and researchers should undertake measures to address.

The acknowledgement of these areas, as well as the issues pertaining to them, acts as an ideal precursor for both identifying and developing solutions within these areas to counter barriers in dementia research. Of particular importance is the training of researchers, which the majority of the issues were associated with data collection, practical issues, researcher characteristics, and a paucity of literature. Researchers are at a disadvantage when it comes to their linguistic and cultural knowledge with regards to ethnic minorities (Brown et al., 2014), and this can be seen across the four barriers, but particularly with data collection.

Previous reviews that identified solutions when conducting recruitment and research with ethnic minorities in mental health research (Waheed et al., 2015) would inform developing tailor‐made solutions aligning with the issues expressed in this review. An example of this is our further research addressing existing gaps regarding the translation and cultural adaptation of cognitive assessments (Mirza, 2016; Mirza, Panagioti, Waheed, & Waheed, 2017). This has been demonstrated through the cultural adaptation of the psychometrically robust cognitive test, the Addenbrooke's Cognitive Examination Version III, for an Urdu‐speaking South Asian population.

This was a multimethod qualitative study detailing how to develop guidelines for culturally adapting any cognitive test and then implementing those guidelines (Mirza, 2016; Mirza, Panagioti, & Waheed, 2018). This was followed by a cultural validation of our Addenbrooke's Cognitive Examination Version III Urdu, demonstrating how to employ cognitive interviews to assess understanding and acceptability of any assessment within a target population (Mirza et al., 2018). These act as solutions to the subthemes “Language issues in assessments,” “Cultural issues in assessments,” and Translation of assessments. This research also presented some solutions to the subtheme General communication issues by referring to cultural sensitivity training the researcher underwent and the measures undertaken to account for South Asian culture and understanding (Mirza et al., 2018).

A further systematic review of all dementia research including ethnic minorities conducted within the UK, with a focus on solutions undertaken by researchers to counter these issues, would also act as a precursor to improving upon existing and developing new solutions. Researchers identified through this review should also be contacted and a qualitative study conducted to investigate further the solutions they proposed and implemented for richer data.

This compilation of recruitment and methodological issues and any companion solutions developed would also act as a check for quality assurance and a guide for future dementia researchers. It would allow for ethnically sound robust dementia research with improvements such as lower rates of false positive and false negative scores through improved and culturally appropriate assessments. It would also improve generalisability due to representative samples enhanced by recruitment and an understanding of heterogeneity in ethnic subgroups and overall reduced cultural bias. This would raise the overall standard of cross‐cultural research with regards to dementia.

An awareness of these recruitment and methodological issues, particularly those associated with the training of researchers, can allow future dementia researchers working with ethnic minorities to account for these issues and plan ahead with regards to the design and execution of their own research.

## 5 CONCLUSIONS

This review is the first systematic review of its kind to identify recruitment and methodological issues within dementia research conducted in the UK that included ethnic minorities. The benefit of this is that we now have a compilation of all the existing issues across the different research stages reported by both participants and researchers themselves. We now have a framework upon which to devise or report solutions for each issue identified, with the interrelated nature of the issues allowing one solution to address multiple issues. These issues, along with developed or identified companion solutions, would act as a guide for future dementia researchers and improve the overall standard of cross‐cultural dementia research.

## AUTHORS' CONTRIBUTIONS

CK, AB, WW, and NM were involved in the designing and running of the search and collated and selected the studies; NM and MWW extracted data, and alongside WW, conducted the qualitative analysis. WW and NM wrote the initial draft, and all authors contributed to the final draft and approved the manuscript.

## CONFLICT OF INTEREST

The authors declare that they have no competing interests.

## References

[mpr1806-bib-0001] Adamson, J. (2001). Awareness and understanding of dementia in African/Caribbean and South Asian families. Health & Social Care in the Community, 9(6), 391–396. 10.1046/j.0966-0410.2001.00321.x 11846818

[mpr1806-bib-0002] Adamson, J. , & Donovan, J. (2005). “Normal disruption”: South Asian and African/Caribbean relatives caring for an older family member in the UK. Social Science & Medicine, 60(1), 37–48. 10.1016/j.socscimed.2004.05.002 15482865

[mpr1806-bib-0003] Adelman, S. , Blanchard, M. , Rait, G. , Leavey, G. , & Livingston, G. (2011). Prevalence of dementia in African–Caribbean compared with UK‐born White older people: two‐stage cross‐sectional study. The British Journal of Psychiatry, 199(2), 119–125. 10.1192/bjp.bp.110.086405 21653946

[mpr1806-bib-0004] Alzheimer's Society (2019). Alzheimer's Society's view on demography. Alzheimer's Society, https://www.alzheimers.org.uk/about-us/policy-and-influencing/what-we-think/demography [accessed 6th May 2019].

[mpr1806-bib-0005] Beaton, D. E. , Bombardier, C. , Guillemin, F. , & Ferraz, M. B. (2000). Guidelines for the process of cross‐cultural adaptation of self‐report measures. Spine, 25(24), 3186–3191. 10.1097/00007632-200012150-00014 11124735

[mpr1806-bib-0006] Beattie, A. , Daker‐White, G. , Gilliard, J. , & Means, R. (2005). “They don't quite fit the way we organise our services”—results from a UK field study of marginalised groups and dementia care. Disability & Society, 20(1), 67–80. 10.1080/0968759042000283647

[mpr1806-bib-0007] Bhatkal, S. , & Shah, A. (2004). The clinical and demographic characteristics of elderly patients of Polish origin newly referred to a geriatric psychiatry service. International Psychogeriatrics, 16(3), 351–360. 10.1017/S1041610204000444 15559758

[mpr1806-bib-0008] Bhatnagar, K. , & Frank, J. (1997). Psychiatric disorders in elderly from the Indian sub‐continent living in Bradford. International Journal of Geriatric Psychiatry, 12(9), 907–912. 10.1002/(SICI)1099-1166(199709)12:9<907::AID-GPS661>3.0.CO;2-8 9309468

[mpr1806-bib-0009] Blakemore, A. , Kenning, C. , Mirza, N. , Daker‐White, G. , Panagioti, M. , & Waheed, W. (2018). Dementia in UK South Asians: A scoping review of the literature. BMJ Open, 8(4), e020290 10.1136/bmjopen-2017-020290 PMC5898329 29654029

[mpr1806-bib-0010] Bowes, A. , & Wilkinson, H. (2003). “We didn't know it would get that bad”: South Asian experiences of dementia and the service response. Health & Social Care in the Community, 11(5), 387–396. 10.1046/j.1365-2524.2003.00440.x 14498835

[mpr1806-bib-0011] Braun, V. , & Clarke, V. (2008). Using thematic analysis in psychology. Qualitative Research in Psychology, 3(2), 77–101.

[mpr1806-bib-0012] Brown, G. , Marshall, M. , Bower, P. , Woodham, A. , & Waheed, W. (2014). Barriers to recruiting ethnic minorities to mental health research: A systematic review. International Journal of Methods in Psychiatric Research, 23(1), 36–48. 10.1002/mpr.1434 24474683 PMC6878438

[mpr1806-bib-0013] Dementia Statistics Hub (2019). Numbers of people in the UK. Alzheimer's Research UK, https://www.dementiastatistics.org/statistics/numbers-of-people-in-the-uk/ [accessed 6th May 2019]

[mpr1806-bib-0014] Department of Health . (2009). Living well with dementia: A national dementia strategy.

[mpr1806-bib-0015] Forbat, L. (2003). Concepts and understandings of dementia by “gatekeepers” and minority ethnic “service users”. Journal of Health Psychology, 8(5), 645–655. 10.1177/13591053030085013 19177723

[mpr1806-bib-0016] Haider, I. , & Shah, A. (2004). A pilot study of behavioural and psychological signs and symptoms of dementia in patients of Indian sub‐continent origin admitted to a dementia day hospital in the United Kingdom. International Journal of Geriatric Psychiatry, 19(12), 1195–1204. 10.1002/gps.1245 15526310

[mpr1806-bib-0017] Hailstone, J. , Mukadam, N. , Owen, T. , Cooper, C. , & Livingston, G. (2017). The development of Attitudes of People from Ethnic Minorities to Help‐Seeking for Dementia (APEND): A questionnaire to measure attitudes to help‐seeking for dementia in people from South Asian backgrounds in the UK. International Journal of Geriatric Psychiatry, 32(3), 288–296. 10.1002/gps.4462 27001896

[mpr1806-bib-0018] Jutlla, K. (2015). The impact of migration experiences and migration identities on the experiences of services and caring for a family member with dementia for Sikhs living in Wolverhampton, UK. Ageing and Society, 35(5), 1032–1054. 10.1017/S0144686X14000658

[mpr1806-bib-0019] Khan, F. , & Tadros, G. (2014). Complexity in cognitive assessment of elderly British minority ethnic groups: Cultural perspective. Dementia, 13(4), 467–482. 10.1177/1471301213475539 24339067

[mpr1806-bib-0020] La Fontaine, J. , Ahuja, J. , Bradbury, N. M. , Phillips, S. , & Oyebode, J. R. (2007). Understanding dementia amongst people in minority ethnic and cultural groups. Journal of Advanced Nursing, 60(6), 605–614. 10.1111/j.1365-2648.2007.04444.x 18039247

[mpr1806-bib-0021] Lakey, L. , Chandaria, K. , Quince, C. , Kane, M. , & Saunders, T. (2012). Dementia 2012: A national challenge (pp. 68–73). London: Alzheimer's Society.

[mpr1806-bib-0022] Lawrence, V. , Murray, J. , Samsi, K. , & Banerjee, S. (2008). Attitudes and support needs of Black Caribbean, south Asian and White British carers of people with dementia in the UK. The British Journal of Psychiatry, 193(3), 240–246. 10.1192/bjp.bp.107.045187 18757985

[mpr1806-bib-0023] Lievesley, N. (2010). The future ageing of the ethnic minority population of England and Wales. London: Runnymede and the Centre for Policy on Ageing.

[mpr1806-bib-0024] Livingston, G. , Leavey, G. , Kitchen, G. , Manela, M. , Sembhi, S. , & Katona, C. (2001). Mental health of migrant elders—the Islington study. The British Journal of Psychiatry, 179(4), 361–366. 10.1192/bjp.179.4.361 11581119

[mpr1806-bib-0026] Mackenzie, J. (2006). Stigma and dementia: East European and South Asian family carers negotiating stigma in the UK. Dementia, 5(2), 233–247. 10.1177/1471301206062252

[mpr1806-bib-0027] McCracken, C. F. , Boneham, M. A. , Copeland, J. R. , Williams, K. E. , Wilson, K. , Scott, A. , … Cleave, N. (1997). Prevalence of dementia and depression among elderly people in black and ethnic minorities. The British Journal of Psychiatry, 171(3), 269–273. 10.1192/bjp.171.3.269 9337983

[mpr1806-bib-0028] Mirza, N. (2016). The translation and cultural adaptation of the Addenbrooke's Cognitive Examination Version III for the British Urdu speaking population. Unpublished thesis. Manchester: The University of Manchester.

[mpr1806-bib-0029] Mirza, N. , Panagioti, M. , Waheed, M. W. , & Waheed, W. (2017). Reporting of the translation and cultural adaptation procedures of the Addenbrooke's Cognitive Examination version III (ACE‐III) and its predecessors: A systematic review. BMC Medical Research Methodology, 17(1), 141 10.1186/s12874-017-0413-6 28903725 PMC5598011

[mpr1806-bib-0030] Mirza, N. , Panagioti, M. , & Waheed, W. (2018). Cultural validation of the Addenbrooke's Cognitive Examination Version III Urdu for the British Urdu‐speaking population: A qualitative assessment using cognitive interviewing. BMJ Open, 8(12), e021057 10.1136/bmjopen-2017-021057 PMC6303692 30552243

[mpr1806-bib-0031] Moher, D. , Liberati, A. , Tetzlaff, J. , & Altman, D. (2009). The PRISMA Group: Preferred reporting items for systematic reviews and meta‐analyses: The PRISMA statement. PLoS Medicine, 6, e1000097 10.1371/journal.pmed1000097 19621072 PMC2707599

[mpr1806-bib-0032] Morgan, T. , & Crowder, R. (2003). Mini mental state examinations in English: Are they suitable for people with dementia who are Welsh speaking? Dementia, 2(2), 267–272. 10.1177/1471301203002002009

[mpr1806-bib-0033] Mukadam, N. , Cooper, C. , Basit, B. , & Livingston, G. (2011). Why do ethnic elders present later to UK dementia services? A qualitative study. International Psychogeriatrics, 23(7), 1070–1077. 10.1017/S1041610211000214 21349212

[mpr1806-bib-0034] Odutoye, K. , & Shah, A. (1999). The characteristics of Indian subcontinent origin elders newly referred to a psychogeriatric service. International Journal of Geriatric Psychiatry, 14(6), 446–453. 10.1002/(SICI)1099-1166(199906)14:6<446::AID-GPS950>3.0.CO;2-L 10398354

[mpr1806-bib-0035] Office for National Statistics, National Records of Scotland, Northern Ireland Statistics and Research Agency (2016). 2011 Census aggregate data. UK Data Service.

[mpr1806-bib-0036] Parveen, S. , Blakey, H. , & Oyebode, J. R. (2018). Evaluation of a carers' information programme culturally adapted for South Asian families. International Journal of Geriatric Psychiatry, 33(2), e199–e204. 10.1002/gps.4768 28766793

[mpr1806-bib-0037] Prince, M. , Knapp, M. , Guerchet, M. , McCrone, P. , Prina, M. , Comas‐Herrera, A. , Wittenberg, E. , et al. (2014). Dementia UK: ‐Overview.

[mpr1806-bib-0038] Purandare, N. , Luthra, V. , Swarbrick, C. , & Burns, A. (2007). Knowledge of dementia among South Asian (Indian) older people in Manchester, UK. International Journal of Geriatric Psychiatry, 22(8), 777–781. 10.1002/gps.1740 17192964

[mpr1806-bib-0039] Rait, G. , Burns, A. , Baldwin, R. , Morley, M. , Chew‐Graham, C. , & St Leger, A. S. (2000). Validating screening instruments for cognitive impairment in older South Asians in the United Kingdom. International Journal of Geriatric Psychiatry, 15(1), 54–62. 10.1002/(SICI)1099-1166(200001)15:1<54::AID-GPS77>3.0.CO;2-C 10637405

[mpr1806-bib-0040] Rait, G. , Morley, M. , Burns, A. , Baldwin, R. , Chew‐Graham, C. , & St Leger, A. S. (2000). Screening for cognitive impairment in older African‐Caribbeans. Psychological Medicine, 30(4), 957–963. 10.1017/S0033291799002305 11037103

[mpr1806-bib-0041] Ramsey, V. , Stevens, S. , Bryan, K. , Binder, J. , & Cockle‐Hearne, J. (2009). Using the Barnes Language Assessment with older ethnic minority groups. International Journal of Geriatric Psychiatry, 24(4), 426–431. 10.1002/gps.2158 19206078

[mpr1806-bib-0042] Redelinghuys, J. , & Shah, A. (1997). The characteristics of ethnic elders from the Indian subcontinent using a geriatric psychiatry service in West London. Aging & Mental Health, 1(3), 243–247. 10.1080/13607869757137

[mpr1806-bib-0043] Regan, J. L. (2016). Ethnic minority, young onset, rare dementia type, depression: A case study of a Muslim male accessing UK dementia health and social care services. Dementia, 15(4), 702–720. 10.1177/1471301214534423 24858552

[mpr1806-bib-0044] Richards, M. , & Abas, M. (1999). Cross cultural approaches to depression and dementia. Royal College of Psychiatrists.

[mpr1806-bib-0045] Richards, M. , Brayne, C. , Dening, T. , Abas, M. , Carter, J. , Price, M. , … Levy, R. (2000). Cognitive function in UK community‐dwelling African Caribbean and white elders: a pilot study. International Journal of Geriatric Psychiatry, 15(7), 621–630. 10.1002/1099-1166(200007)15:7<621::AID-GPS164>3.0.CO;2-4 10918343

[mpr1806-bib-0046] Ryan, G. W. , & Bernard, H. R. (2003). Techniques to identify themes. Field Methods, 15(1), 85–109. 10.1177/1525822X02239569

[mpr1806-bib-0048] Seabrooke, V. , & Milne, A. (2004). Culture and care in dementia: A study of the Asian community in north West Kent. Northfleet: Alzheimer's and Dementia Support Services/Mental Health Foundation.

[mpr1806-bib-0049] Shah, A. (1999). Difficulties experienced by a Gujarati geriatric psychiatrist in interviewing Gujarati elders in Gujarati. International Journal of Geriatric Psychiatry, 14(12), 1072–1074. 10.1002/(SICI)1099-1166(199912)14:12<1072::AID-GPS93>3.0.CO;2-W 10607975

[mpr1806-bib-0050] Shah, A. , Lindesay, J. , & Jagger, C. (1998). Is the diagnosis of dementia stable over time among elderly immigrant Gujaratis in the United Kingdom (Leicester)? International Journal of Geriatric Psychiatry, 13(7), 440–444. 10.1002/(SICI)1099-1166(199807)13:7<440::AID-GPS793>3.0.CO;2-W 9695031

[mpr1806-bib-0051] Speer, S. A. , & McPhillips, R. (2013). Patients' perspectives on psychiatric consultations in the gender identity clinic: implications for patient‐centered communication. Patient Education and Counseling, 91, 385–391. 10.1016/j.pec.2012.12.009 23369376

[mpr1806-bib-0052] Stewart, R. , Richards, M. , Brayne, C. , & Mann, A. (2001). Cognitive function in UK community‐dwelling African Caribbean elders: Normative data for a test battery. International Journal of Geriatric Psychiatry, 16(5), 518–527. 10.1002/gps.384 11376469

[mpr1806-bib-0053] Stewart, R. , Russ, C. , Richards, M. , Brayne, C. , Lovestone, S. , & Mann, A. (2001). Apolipoprotein E genotype, vascular risk and early cognitive impairment in an African Caribbean population. Dementia and Geriatric Cognitive Disorders, 12(4), 251–256. 10.1159/000051267 11351136

[mpr1806-bib-0054] Turner, S. , Christie, A. , & Haworth, E. (2005). South Asian and white older people and dementia: A qualitative study of knowledge and attitudes. Diversity & Equality in Health and Care, 2(3).

[mpr1806-bib-0055] Uppal, G. K. , Bonas, S. , & Philpott, H. (2014). Understanding and awareness of dementia in the Sikh community. Mental Health, Religion and Culture, 17(4), 400–414. 10.1080/13674676.2013.816941

[mpr1806-bib-0056] Waheed, W. , Hughes‐Morley, A. , Woodham, A. , Allen, G. , & Bower, P. (2015). Overcoming barriers to recruiting ethnic minorities to mental health research: A typology of recruitment strategies. BMC Psychiatry, 15(1), 101. 25934297 10.1186/s12888-015-0484-z PMC4436137

[mpr1806-bib-0057] Weimer, D. L. , & Sager, M. A. (2009). Early identification and treatment of Alzheimer's disease: Social and fiscal outcomes. Alzheimer's & Dementia: The Journal of the Alzheimer's Association, 5(3), 215–226. 10.1016/j.jalz.2009.01.028 PMC2785909 19362885

[mpr1806-bib-0058] Wilkinson, H. , Bowes, A. , & Rodrigues, A. (2003). Innovative methodologies—Can we learn from including people with dementia from South Asian communities? Research Policy and Planning, 21(2), 43–54.

[mpr1806-bib-0059] Wohland, P. , Rees, P. , Norman, P. , Boden, P. , & Jasinska, M. (2010). Ethnic population projections for the UK and local areas, 2001–2051. University of Leeds http://www.geog.leeds.ac.uk/fileadmin/downloads/school/research/projects/migrants/WP_ETH_POP_ PROJECTIONS. pdf [accessed 10 April 2011].

